# Development of a cross-protective common cold coronavirus vaccine

**DOI:** 10.1128/jvi.01526-25

**Published:** 2025-10-22

**Authors:** Tanushree Dangi, Shiyi Li, Pablo Penaloza-MacMaster

**Affiliations:** 1 Center for Virology and Vaccine Research, Beth Israel Deaconess Medical Center, Harvard Medical School 1811 , Boston, Massachusetts, USA; 2 Department of Microbiology-Immunology, Feinberg School of Medicine, Northwestern University 12244, Chicago, Illinois, USA; Loyola University Chicago - Health Sciences Campus, Maywood, Illinois, USA

**Keywords:** coronavirus, HCoV-OC43, vaccines, cross-protective immunity

## Abstract

Common cold coronaviruses, such as OC43 and HKU1, typically cause mild respiratory infections in healthy people. However, they can lead to severe illness in high-risk groups, including immunocompromised individuals and older adults. Currently, there is no clinically approved vaccine to prevent infection by common cold coronaviruses. Here, we developed an mRNA vaccine expressing a stabilized spike protein derived from OC43 coronavirus and tested its efficacy in different challenge models in C57BL/6 mice. This novel OC43 vaccine elicited OC43-specific immune responses, as well as cross-reactive immune response against other embecoviruses, including HKU1 and mouse hepatitis virus (MHV-A59). Interestingly, this OC43 vaccine protected mice not only against a lethal OC43 infection but also against a distant embecovirus, MHV-A59. These findings provide insights for the development of common cold coronavirus vaccines, demonstrating their potential to protect against various coronaviruses.

**IMPORTANCE:**

Human coronaviruses like OC43 cause disease in vulnerable populations, yet no approved vaccines exist. We developed an mRNA vaccine targeting the OC43 spike protein that protects mice not only against homologous OC43 challenges but also against the distantly related embecovirus MHV-A59. These findings demonstrate the feasibility of a single vaccine conferring broad protection across multiple coronaviruses within the same subgenus, advancing strategies for pan-coronavirus vaccine development.

## INTRODUCTION

Severe acute respiratory syndrome coronavirus 2 (SARS-CoV-2) has resulted in over 7 million deaths globally. Despite this high number of deaths, the rapid development of SARS-CoV-2 vaccines reduced the death toll of the pandemic. In addition to SARS-CoV-2, several endemic coronaviruses like OC43, 229E, HKU1, and NL63 circulate within the human population, causing frequent re-infections. While these endemic coronaviruses typically cause mild to moderate upper respiratory infections, they can also lead to severe conditions, pneumonia, and bronchitis, particularly in the elderly and immunocompromised individuals (1–6). Among these, the human common cold coronavirus, OC43, causes frequent respiratory infections worldwide, but no effective vaccine is currently available.

The widespread circulation of OC43 poses public health concerns due to its propensity for mutation and potential recombination with other coronaviruses. OC43 can cause a burden on healthcare systems, resulting in economic losses. The high incidence of OC43 re-infections further highlights the need for effective vaccines (7–10). Addressing these challenges with an effective vaccine would not only improve overall health in the population but also have the potential to alleviate the economic burden associated with recurrent coronavirus infections. Here, we develop an mRNA vaccine encoding a stabilized OC43 spike protein. Our results demonstrate that this vaccine is immunogenic and highly protective not only against OC43, but also a distant embecovirus.

## RESULTS

### Design of a novel mRNA-LNP vaccine encoding HCoV-OC43 spike glycoprotein (mRNA-OC43)

**Fig 1:**
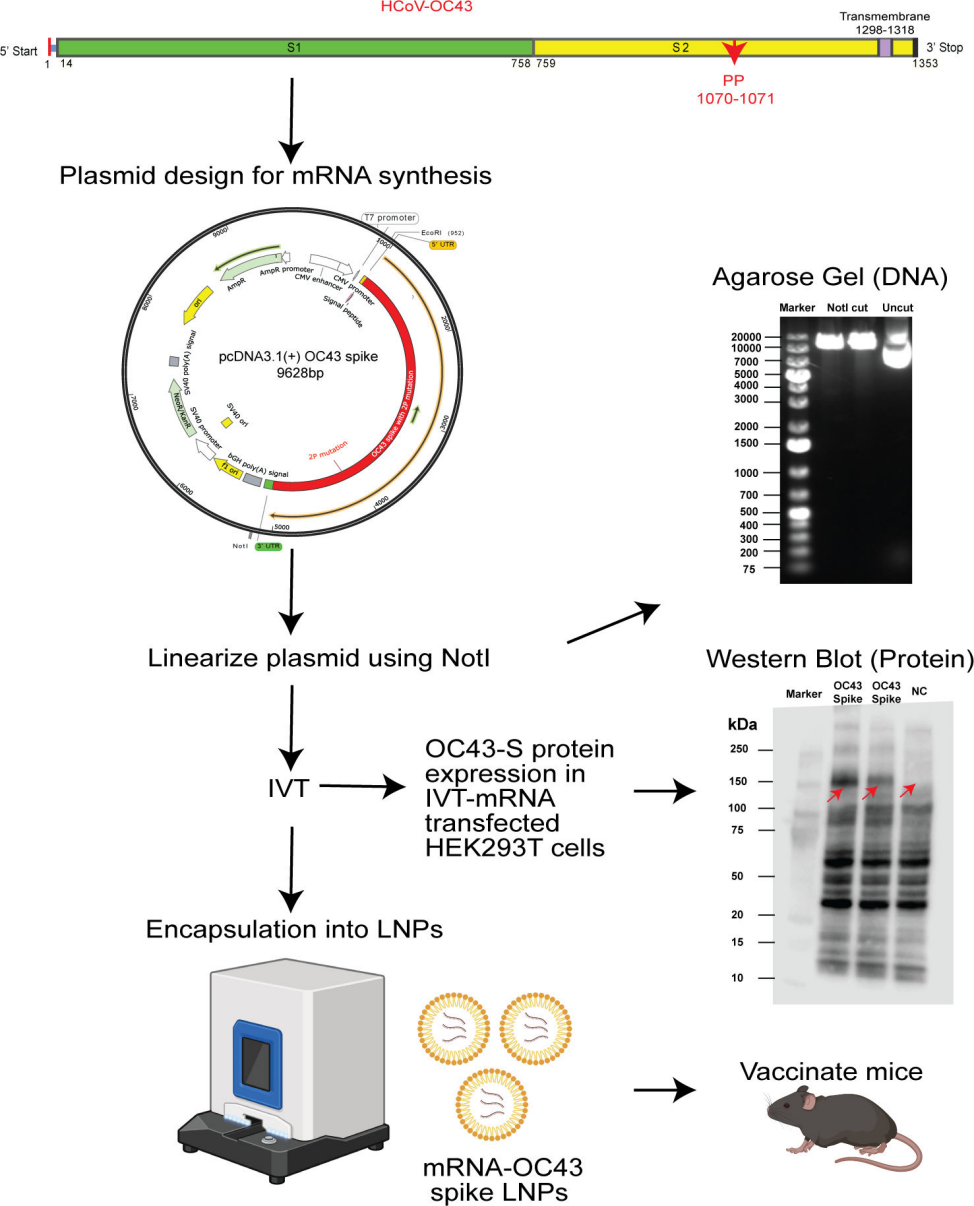
Formulation strategy of mRNA-based common cold coronavirus vaccine encoding OC43-spike protein. The spike gene sequence of the OC43 was modified by inserting two proline mutations in the S2 subdomain and was codon-optimized for mouse. pcDNA3.1(+) plasmid was constructed by incorporating modified OC43-spike gene. Before proceeding with the IVT reaction, the plasmid construct was linearized using NotI restriction digestion, and its size was confirmed by agarose gel electrophoresis. mRNA was transcribed *in vitro* (IVT-mRNA) using a plasmid construct and incorporated with 0 cap and poly-A tail at 5′ and 3′ ends of transcribed mRNA. The expression of the OC43 spike gene was tested in HEK293T cells transfected with mRNA-OC43 and by analyzing the transfected cell lysate in Western blot. The vaccine was prepared by encapsulating IVT-mRNA into lipid nanoparticles utilizing the Nanoassemblr platform.

We developed an mRNA vaccine encoding the full-length spike glycoprotein of the OC43 coronavirus. We utilized the spike protein sequence from NCBI (AAA03055.1) and introduced two proline mutations at 1070 and 1071 amino acid positions (replaced with A and L amino acids) in the S2 domain to stabilize the spike protein in its pre-fusion state. A pcDNA3.1 (+) plasmid construct was designed by incorporating the codon-optimized spike gene sequence flanked with untranslated regions (UTRs) at 3′ and 5′ ends, and a T7 RNA polymerase site before the 3′ end of coding sequence. Expression of OC43 spike glycoprotein was confirmed by transfecting HEK293T cells with the corresponding mRNA followed by Western Blot analysis to validate protein expression (Fig. 1). mRNA molecules were then encapsulated into a lipid nanoparticle using a Nanoassemblr.

### Immunogenicity and protective efficacy of mRNA-OC43 following homologous OC43 challenge

**Fig 2:**
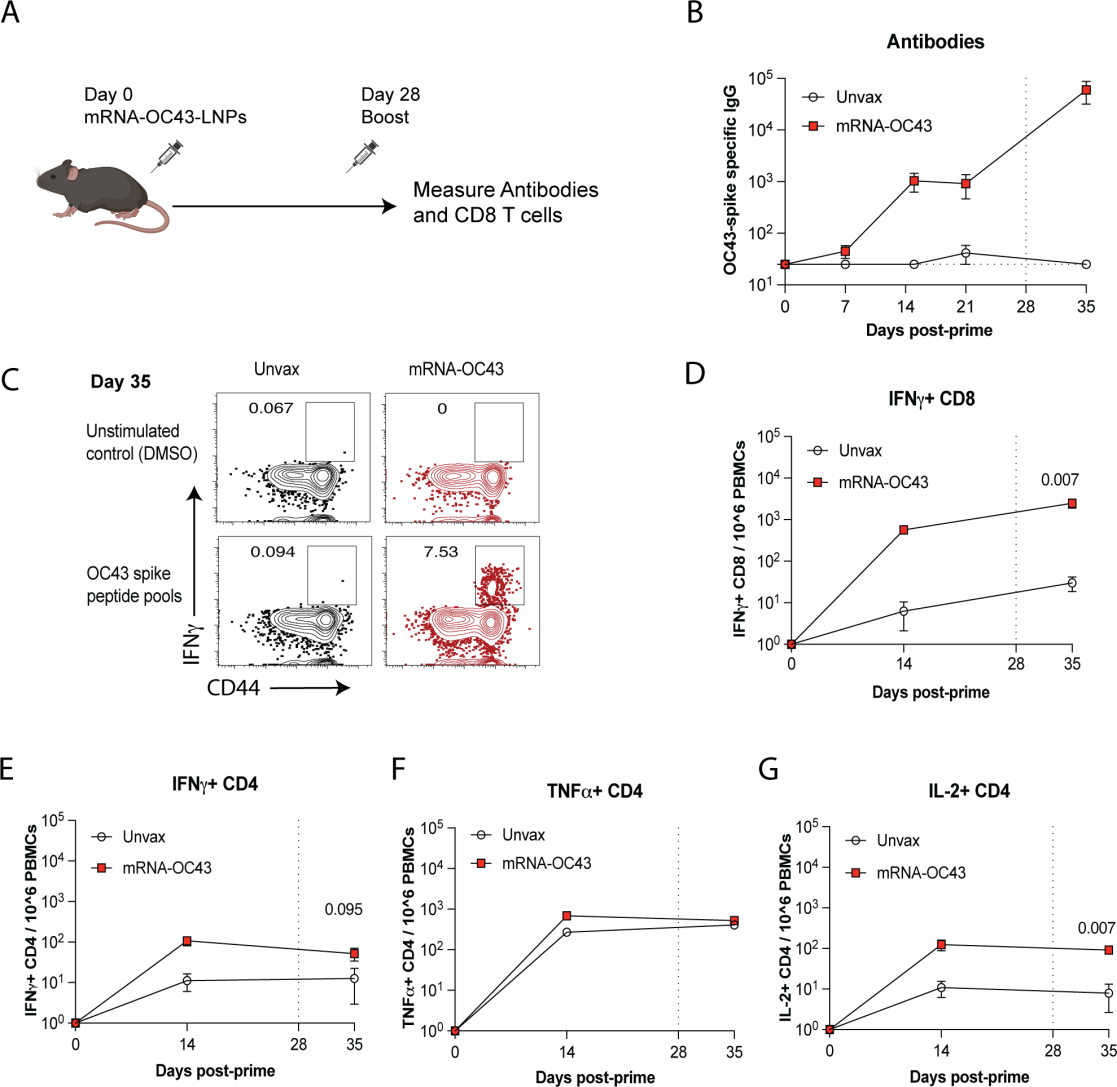
mRNA-OC43 elicits antibody and T cell responses. (**A**) Experimental outline representing the immunization scheme in C57BL/6 mice. Mice were primed and boosted intramuscularly with 3 µg of OC43 spike mRNA vaccine. (**B**) Summary of OC43-specific antibody responses in sera. Vertical dashed line indicates time of boosting. The horizontal dashed line represents the limit of detection. (C to G) At days 15 and 35 post-vaccination, PBMCs were stimulated with overlapping OC43 spike peptide pools for 5 hours at 37°C in the presence of GolgiStop and GolgiPlug to evaluate OC43-specific CD8+ and CD4+ T-cell responses. As a negative control, cells were stimulated with vehicle control (DMSO) in the presence of GolgiStop and GolgiPlug. (**C**) Representative FACS plots showing the frequencies of OC43-specific CD8 T cells expressing IFNγ in PBMCs at day 35 post-vaccination. (**D**) Summary of OC43-specific CD8 T cells that express IFNγ in PBMCs. (**E**) Summary of OC43-specific CD4 T cells that express IFNγ in PBMCs. (**F**) Summary of OC43-specific CD4 T cells that express TNFα in PBMCs. (**G**) Summary of OC43-specific CD4 T cells that express IL-2 in PBMCs. Data are from two experiments, *n* = 5 mice per group. Indicated *P* values were determined by a nonparametric Mann-Whitney U test (unpaired *t*-test). Dashed lines indicate the limit of detection. Error bars represent SEM.

To evaluate immunogenicity, we first immunized C57BL/6 mice intramuscularly with 3 µg of mRNA-OC43 (Fig. 2A). After vaccination, we measured antibody and T cell responses in plasma, using enzyme-linked immunosorbent assay (ELISA) with OC43 spike as coating antigen, and intracellular cytokine assay (ICS) using overlapping OC43 spike peptide pools. The mRNA-OC43 vaccine elicited potent antibody responses to the OC43 spike protein (Fig. 2B). Moreover, the vaccine elicited CD8 and CD4 T-cell responses by ICS (Fig. 2C through G).

**Fig 3:**
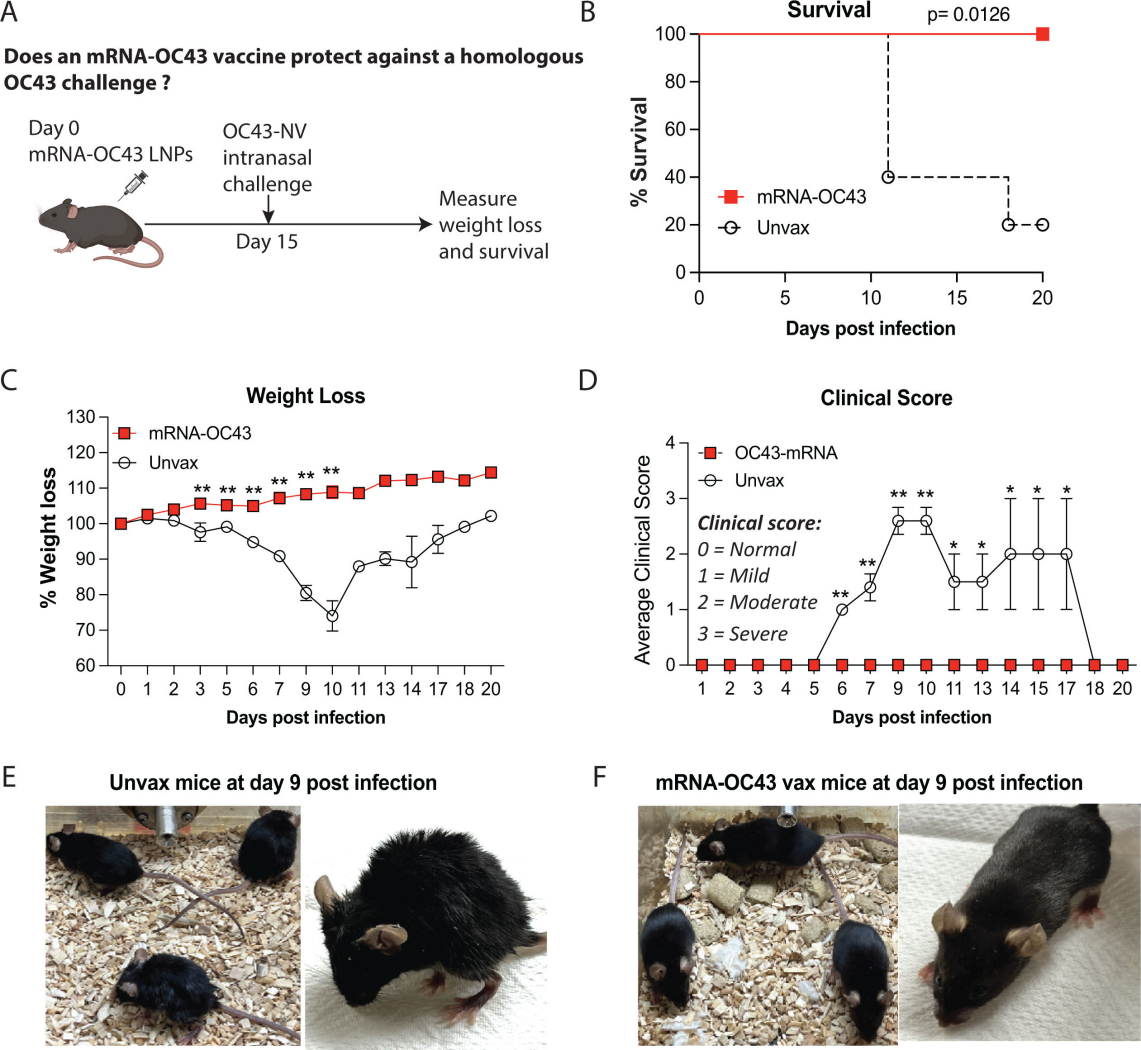
mRNA-OC43 vaccine protects mice from homologous OC43 infection. (**A**) Experimental outline for evaluating whether mRNA-OC43 vaccine protects mice against homologous OC43 infections. C57BL/6 mice were immunized with 3 μg of mRNA-OC43 vaccine intramuscularly. Control mice received PBS. At 2 weeks following vaccination, 50 μL of OC43 NV strain (1 × 10¹⁰ genome copies) was intranasally inoculated into mice. Mice were monitored over 20 days for weight changes, mortality, and clinical signs of disease including hunched posture, ruffled fur, lethargy, and labored breathing. (**B**) Survival. (**C**) Weight loss. (**D**) Clinical score. Weight loss was calculated in terms of percent of original weight. Clinical scores were assigned on a scale of 0–3 in each category, where clinical scores 0, 1, 2, and 3 represent normal healthy, mild, moderate, and severe clinical signs/symptoms, respectively (see the details of the disease score in methods). Daily scores were averaged. (**E**) Representative images of unvaccinated mice at day nine post-OC43 infection. Mice showed severe clinical pathology and had to be euthanized. (**F**) Representative images of mRNA-OC43 immunized mice at day 9 post-OC43 infection. Mice were active with smooth coat appearance. Clinical scores and weight were compared using multiple Student’s *t*-tests with Holm-Sidak multiple comparison correction. Survival curves were compared using the log-rank Mantel-Cox comparison test. Significant differences compared to control are indicated *P ≤ 0.05, **P ≤ 0.01. Data are from one experiment, including 5 mice per group. Error bars represent SEM.

To examine vaccine efficacy, mice were challenged intranasally with 50 µL of OC43 neurovirulent (NV) strain (1 × 10^10^ genome copies) at week 2 post-vaccination. Mice were monitored daily for any changes in clinical signs or symptoms, body mass, and mortality (Fig. 3A). Upon OC43 challenge, unvaccinated mice exhibited severe weight loss, severe clinical pathology, and showed only 20% survival (Fig. 3B through E). In contrast, mice that received the OC43 vaccine showed no weight loss and 100% survival, with no clinical signs of disease (Fig. 3F).

### Protective efficacy of mRNA-OC43 following a heterologous coronavirus challenge

**Fig 4:**
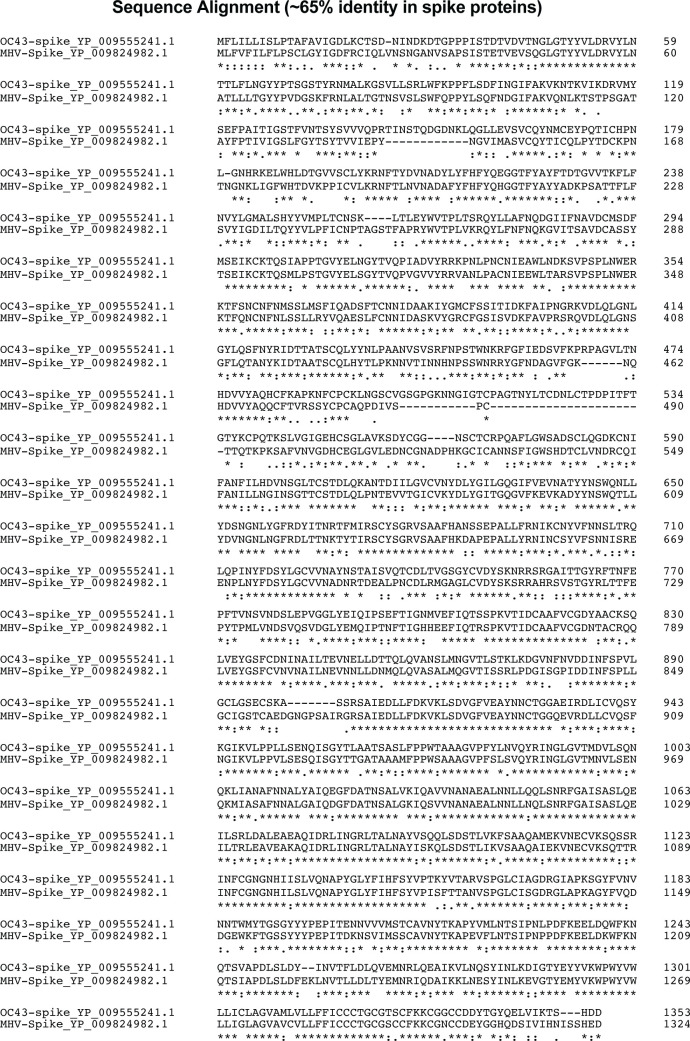
Sequence identity in spike glycoproteins of HCoV-OC43 and MHV-A59. Amino acid alignment of spike protein sequences of OC43 and MHV-A59 representing a 65% sequence identity in spike protein. Asterisks indicate identical residues; colons represent conserved changes, and blank spaces denote non-conserved substitutions.

Further, we interrogated whether the mRNA-OC43 vaccine could cross-protect against another embecovirus (MHV-A59). MHV-A59 is a well-studied mouse virus (11–13). OC43 and MHV-A59 share only ~65% sequence identity in their spike proteins, rendering MHV-A59 a stringent challenge model to examine cross-protection by our mRNA-OC43 vaccine (Fig. 4). While MHV-A59 is not considered a significant threat to humans, it serves as a useful proof-of-principle model to evaluate the protective breadth of our OC43 vaccine.

**Fig 5:**
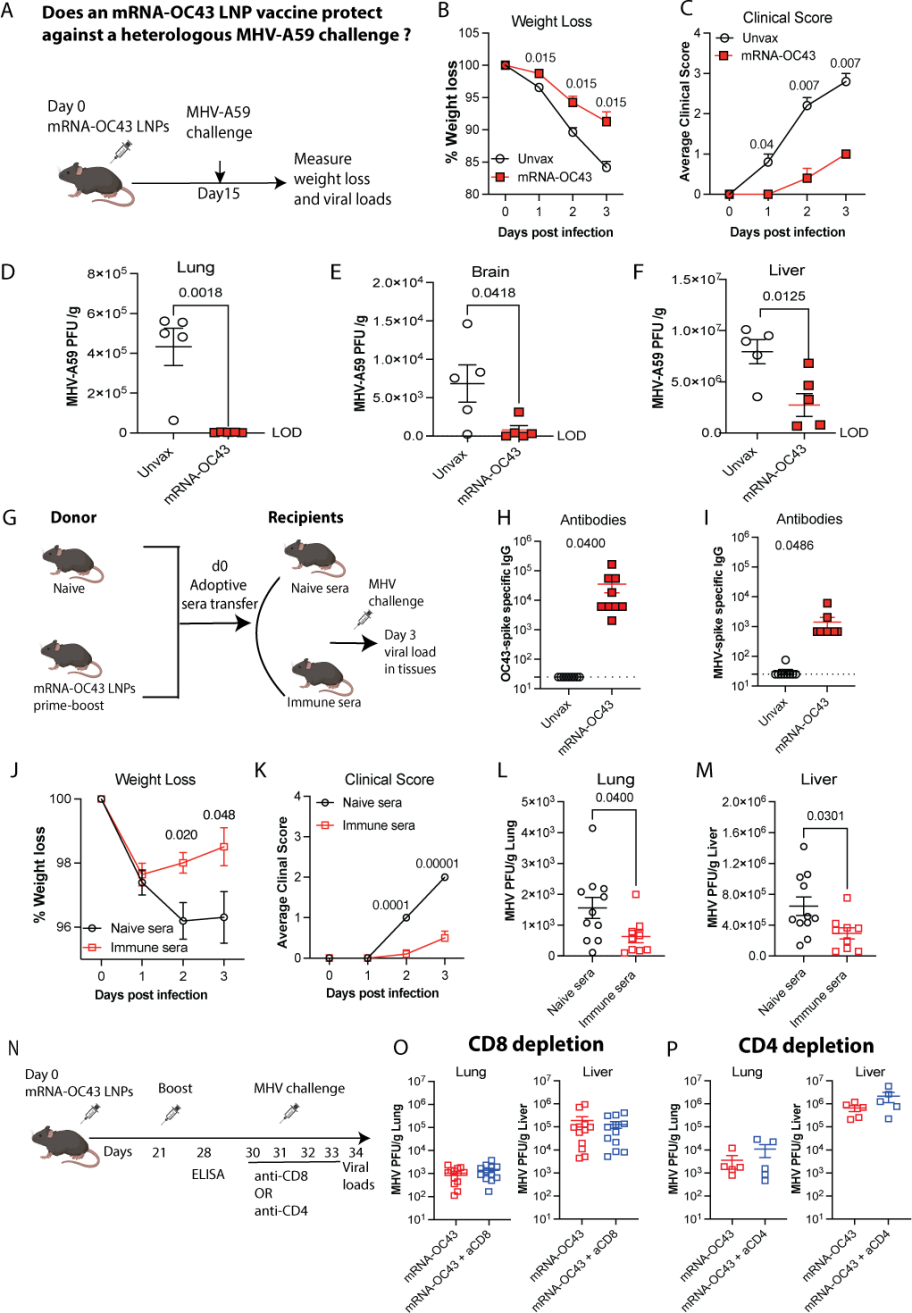
mRNA-OC43 vaccine protects mice against heterologous MHV-A59 infection. (**A**) Experimental outline for investigating the efficacy of mRNA-OC43 vaccine in C57BL/6 mice upon MHV-A59 challenge. Mice were immunized with 3 μg of mRNA-OC43 vaccine intramuscularly. Control mice received PBS. At day 15 following vaccination, all mice were inoculated with 500 μL of MHV-A59 (2 × 10⁶ PFU) via i.p. route. Body weight and clinical signs of disease (hunched posture, ruffled fur, lethargy, and labored breathing) were measured daily over 3 days. Lung, brain, and liver were collected on day 3 post-infection to assess viral load by plaque assay. (**B**) Weight loss was calculated in terms of percent of original weight. (**C**) Clinical score. Clinical scores were assigned on a scale of 0–3 in each category, where clinical scores 0, 1, 2, and 3 represent normal healthy, mild, moderate, and severe clinical signs/symptoms, respectively (see methods for details). Daily scores were averaged. Clinical scores and weight were compared using multiple Student’s *t*-tests with Holm-Sidak multiple comparison correction. (**D**) Viral load in the lung. LOD = 29 PFU/g. (**E**) Viral load in the brain. LOD = 11 PFU/g. (**F**) Viral load in the liver. LOD = 5 PFU/g. (**G**) Schematic layout of adoptive plasma transfer experiment: Donor mice were primed and boosted with 3 µg of mRNA-OC43 vaccine at 4-week interval and harvested plasma on day 35. Then, 800 µL of pooled plasma per mouse was adoptively transferred into recipient mice via i.p. injection, followed by MHV-A59 challenge on the next day. Control mice received naïve plasma. Body weight and clinical signs were measured for three consecutive days. (**H**) OC43 spike-specific antibody responses detected by ELISA in donor plasma on day 35 post-prime. (**I**) Cross-reactive MHV spike-specific antibody responses detected by ELISA in donor plasma on day 35 post-prime (lysates from HEK293T cells transfected with DNA encoding MHV spike were used as coating antigen). Plasma from five donor mice was used in these adoptive transfer experiments. (**J**) Weight loss of recipient mice following MHV-A59 challenge. (**K**) Clinical score. (**L**) Viral load in lung. LOD = 36 PFU/g. (**M**) Viral load in liver. LOD = 5 PFU/g. (**N**) Schematic of the T-cell depletion experiments. Vaccinated and unvaccinated control mice were administered with CD8 or CD4 depleting antibodies for four consecutive days, starting one day before MHV-A59 challenge and continuing through day 3 post-infection. Viral loads in the lungs and liver were measured on day 3 post-infection. (**O**) Viral load in tissues of CD8-depleted mice. (**P**) Viral load in tissues of CD4-depleted mice. Parametric Student’s *t*-tests were used to calculate *P* values, except for B–C and J–K, where nonparametric Mann–Whitney U tests were employed. Data are from one experiment (A–F and *P*) or two experiments (G–O), with *n* = 5 mice per group. All data are shown. Dashed lines indicate the limit of detection. Error bars represent SEM.

To interrogate the cross-protective efficacy of the OC43 vaccine, mice were immunized intramuscularly with 3 µg of the mRNA-OC43 vaccine. After 2 weeks post-vaccination, mice were challenged intraperitoneally (i.p.) with 2 × 10^6^ plaque forming units (PFU) of MHV-A59 (Fig. 5A). All mice experienced weight loss following infection, but the mice that were vaccinated with the mRNA-OC43 vaccine exhibited significantly less weight loss compared with control (Fig. 5B). Further, the clinical signs were significantly milder in mRNA-OC43 vaccinated mice (Fig. 5C). MHV infection is very transient in C57BL/6 mice. In our hands, all mice resolve acute MHV infection after 5–7 days following intraperitoneal challenge without succumbing to infection, regardless of immunization status. Therefore, we selected day 3 post-challenge as the optimal time for evaluating viral loads in vaccinated mice. All mice were sacrificed at day 3 post-infection for assessing viral load in tissues. Importantly, the mRNA-OC43 vaccinated mice showed enhanced viral control in lung, brain, and liver (Fig. 5D through F). These data suggest that the mRNA-OC43 vaccine provides cross-protection against a heterologous coronavirus with only a 65% antigen match.

### Humoral responses elicited by mRNA-OC43 confer cross-protection against MHV-A59

Vaccine protection is typically mediated by humoral and cellular responses. To specifically assess the role of humoral protection, we performed a passive immunization study (Fig. 5G). First, we immunized C57BL/6 mice with the mRNA-OC43 vaccine on days 0 and 21 and then collected immune plasma on day 35. Prior to adoptive transfer, antibody titers specific to the OC43 spike antigen were confirmed in immune plasma using ELISA (Fig. 5H). These mRNA-OC43-immune plasma also exhibited cross-reactivity against an MHV-spike antigen encoded by HEK293T cell lysate (Fig. 5I). Each recipient mouse received 800 µL of pooled plasma via i.p. injection. Control mice received plasma from naïve animals. On the following day, all recipient mice were challenged i.p. with 2 × 10^6^ PFU of MHV-A59. Mice were monitored for three consecutive days for weight loss and clinical severity. We determined viral load in tissues on day 3 after infection. Notably, mice that received immune plasma exhibited reduced weight loss, milder clinical symptoms, and significantly lower viral loads in the lungs (2.5-fold reduction) and liver (2.2-fold reduction) (Fig. 5J through M), indicating that antibodies elicited by the mRNA-OC43 vaccine provide cross-protection against MHV-A59 infection.

Next, we investigated whether T cells elicited by the mRNA-OC43 vaccine contribute to the control of MHV-A59 (Fig. 5N). To assess the role of CD8^+^ T cells, we depleted them at the time of infection, using depleting antibodies. CD8^+^ T cell depletion had no significant effect on viral control (Fig. 5O). Similarly, in separate experiments, we depleted CD4^+^ T cells to determine whether they impaired vaccine protection. CD4^+^ T cell depletion also did not impair vaccine protection (Fig. 5P). These data suggest that T cells are dispensable for mRNA-OC43 vaccine cross-protection against MHV, although it is important to clarify that depleting antibodies may not fully deplete all T cells in tissues (14). Moreover, functional compensation between CD4 and CD8 T cells remains a possible explanation.

### mRNA-MHV vaccine confers heterologous protection against OC43

**Fig 6:**
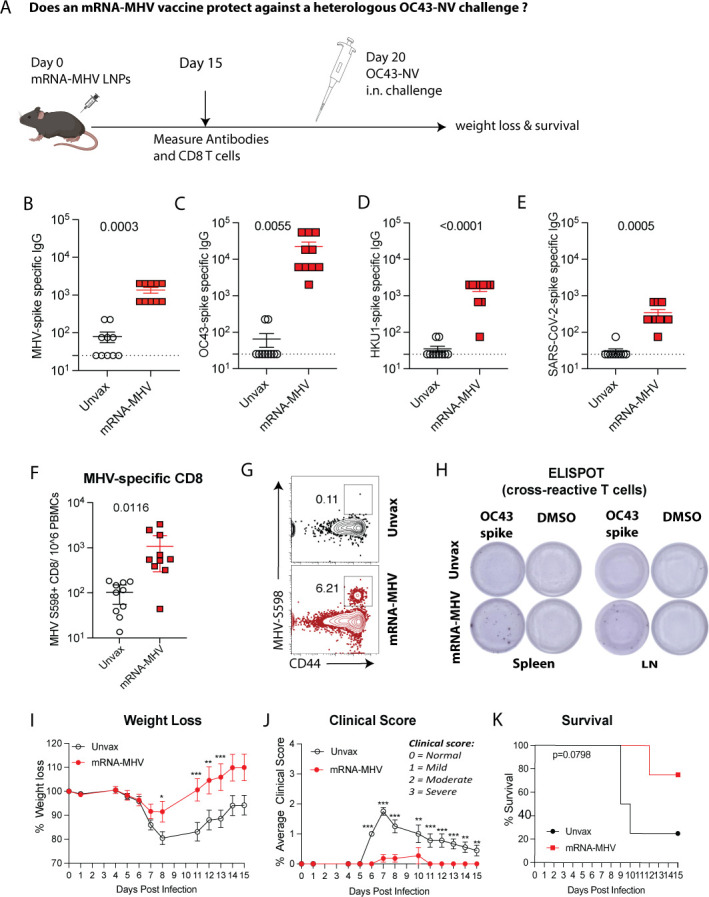
An mRNA-MHV vaccine provides cross-protection against heterologous OC43 infection. (**A**) Schematic representation of experiment investigating the cross-immune protection provided by mRNA-MHV vaccine against heterologous OC43 infection in C57BL/6 mice. Mice were primed with 5 μg of mRNA-MHV vaccine intramuscularly. Control mice received PBS. On day 20 after vaccination, mice were intranasally inoculated with 50 μL of OC43 (1 × 10¹⁰ genome copies). Body weight and clinical signs of disease (hunched posture, ruffled fur, lethargy, and labored breathing) were measured daily over 2 weeks. Antibody responses were tested at day 15 post-vaccination by ELISA. (**B**) Summary of MHV spike-specific antibodies (cell lysate from transfected HEK293T cells). (**C**) Summary of OC43-spike-specific cross-reactive antibodies. (**D**) Summary of HKU1-spike-specific cross-reactive antibodies. (**E**) Summary of SARS-CoV-2-spike-specific cross-reactive antibodies. (**F**) Summary of MHV-spike (K^b^S598) specific CD8^+^ T cells in PBMCs at day 15 post vaccination. (**G**) Representative FACS plots of MHV-spike (K^b^S598) specific CD8^+^ T cells in PBMCs. (**H**) Representative ELISpot showing the cross-reactive OC43 spike-specific IFNγ T cell responses in spleen and draining lymph nodes (LNs). DMSO was used as a negative control. (**I**) Weight loss was calculated in terms of percent of original weight. (**J**) Clinical scores were assigned on a scale of 0–3 in each category, where clinical scores 0, 1, 2, and 3 represent normal healthy, mild, moderate, and severe clinical signs/symptoms, respectively (see methods for details). Daily scores were averaged. (**K**) Survival. Parametric Student’s *t*-tests were used to calculate *P* values for B–F. Clinical scores and weight were compared using multiple Student’s *t*-test, and indicated *P* values were determined by nonparametric Mann-Whitney U test (unpaired *t*-test). Significant differences compared with control are indicated *P ≤ 0.05, **P ≤ 0.01, ***P ≤ 0.001. Survival curves were compared using the log-rank Mantel-Cox comparison test. Data are from two experiments, with *n* = 5 mice per group. All data are shown. Dashed lines indicate the limit of detection. Error bars represent SEM.

We have shown that an mRNA-OC43 vaccine confers heterologous protections against MHV. We also performed the “inverse” vaccination challenge study. Mice were immunized intramuscularly with an mRNA-MHV vaccine followed by a lethal challenge with OC43 (Fig. 6A). On day 15 after vaccination, antibody and T cell responses were measured. As expected, the mRNA-MHV vaccine induced antibody responses against its matched antigen, the MHV spike protein (Fig. 6B). Interestingly, this mRNA-MHV vaccine also elicited cross-reactive antibody responses against other coronaviruses, including OC43, HKU1, and SARS-CoV-2 (Fig. 6C through E). This vaccine elicited MHV-specific CD8^+^ T cell responses (K^b^S598) (Fig. 6F and G), and also cross-reactive OC43-specific CD8^+^ T cell responses by ELISpot assays (Fig. 6H).

To evaluate cross-protective efficacy by the MHV vaccine, mice were intranasally challenged with OC43 (NV strain) 3 weeks after vaccination and then monitored for weight loss and clinical score. Following an OC43 challenge, unvaccinated mice exhibited more significant weight loss and worse disease compared with vaccinated mice (Fig. 6I and J). There was also a pattern of improved survival with the mRNA-MHV vaccine relative to control, but the difference was not statistically significant (Fig. 6K). These results suggest that an mRNA-MHV vaccine confers partial protection against a distant OC43 coronavirus.

## DISCUSSION

There are four endemic human coronaviruses that typically cause mild respiratory infections. These include two alphacoronaviruses (HCoV-229E and HCoV-NL63) and two betacoronaviruses (HCoV-OC43 and HKU1), which are both part of the embecovirus sublineage. In this study, we focused on OC43, given the availability of a mouse model and the fact that it accounts for a great fraction of common cold coronavirus infections in humans (15, 16). OC43 belongs to the betacoronavirus genus, alongside SARS-CoV-2, SARS-CoV, and MERS-CoV, which were responsible for outbreaks in 2019, 2003, and 2012, respectively.

While previous studies have shown that coronavirus vaccines can generate cross-reactive antibodies against endemic coronaviruses, it remains unclear whether these antibodies are cross-protective *in vivo* (17–20). Building on this, we hypothesized that an mRNA vaccine targeting the human common cold coronavirus OC43, for which no effective vaccine currently exists, could also confer cross-protection against other coronaviruses. To test this hypothesis, we developed a novel mRNA vaccine encoding a stabilized OC43 spike protein and assessed its immunogenicity and protective efficacy *in vivo*. This mRNA-OC43 vaccine elicited adaptive immune responses to OC43 and conferred protection against OC43 infection, which was expected given that the vaccine antigen was matched to the challenge antigen. However, an interesting finding was that the mRNA-OC43 vaccine also provided cross-protection against MHV-A59, despite OC43 and MHV-A59 having only 65% identity in their spike proteins. Notably, antibodies induced by the OC43 vaccine exhibited cross-reactivity with MHV-spike. This antibody-mediated cross-reactivity was further supported by adoptive plasma transfer experiments, which showed that antibodies elicited by the mRNA-OC43 vaccine conferred protection to mice against MHV infection, underscoring the importance of humoral immunity in cross-protection. Interestingly, unlike previous reports that highlight a key role for virus-specific CD8^+^ and CD4^+^ T cells in cross-protection (21–24), our model did not reveal a cross-protective role for these T cell subsets in the context of MHV infection. “This could be because T cell-depleting antibodies do not reach all tissues, rendering some tissue-resident memory T cells (Trms) undepleted” (14). In addition, it is possible that depletion of CD8 T cells results in functional compensation by CD4 T cells, and vice versa.

Thus, the depleting antibodies used in our study may have effectively eliminated T cells in the blood, but not in tissues, potentially obscuring their contribution to cross-protection.

To further substantiate our findings, we immunized mice with an mRNA vaccine encoding the MHV spike protein and evaluated its protective efficacy against OC43 infection. This also conferred cross-protection, as demonstrated by reduced weight loss, milder clinical symptoms, and increased survival rates in vaccinated animals. Interestingly, this mRNA-MHV vaccine elicited cross-binding antibody responses to multiple betacoronaviruses, including OC43, HKU1, and SARS-CoV-2, as well as cross-reactive T-cell responses targeting the OC43 spike protein. These results suggest that vaccines targeting a single coronavirus strain can confer broad protection against other coronaviruses from the same embecovirus subgenus. These data may be important for improving vaccine preparedness against circulating and emerging coronaviruses, for example, by pre-emptively developing vaccines to representative coronaviruses from each subgenus.

## MATERIALS AND METHODS

### Mice and Immunizations

Six - to 8-week-old C57BL/6 mice were used. Mice were purchased from Jackson laboratories (approximately half males and half females). Mice were immunized intramuscularly with mRNA-LNPs (made in-house) diluted in sterile PBS. Mice were housed at Northwestern University’s Center for Comparative Medicine (CCM).

### Synthesis of modified mRNA

We synthesized mRNA vaccines encoding for the codon-optimized OC43 spike protein from HCoV-OC43 (accession number AAA03055.1) and codon-optimized MHV-spike protein from MHV-JHM strain (accession number YP_209233.1). For *in vitro* transcription of mRNA (IVT-mRNA), plasmid constructs were designed by incorporating codon-optimized immunogens (OC43-spike or MHV-spike), UTRs, and phase T7 RNA polymerase promoter and purchased from Genscript. The sequences of the 5′- and −3′-UTRs were identical to those used in a previous publication (20). Modified nucleotide pseudouridine-5′-triphosphate (ΨTP), along with canonical nucleotides ATP, CTP, and GTP (CellScript, Cat. No. ICTY110510), was used to synthesize nucleoside-modified IVT-mRNA from the plasmid construct. To enhance mRNA stability, an N7-methylguanosine cap (Cap 1, m⁷G) was added to the 5′ end, and a ~ 150-nucleotide poly(A) tail was incorporated at the 3′ end using CellScript Capping and Tailing Kits (Cat. Nos. SCCS1710 and PAP5104H). The IVT-mRNA was purified via ammonium acetate precipitation and quantified using a NanoDrop ONE spectrophotometer (Thermo Scientific). To evaluate protein expression, purified mRNA was transfected into female human embryonic kidney (HEK) 293T cells using the TransIT-mRNA Transfection Kit (Mirus, Cat. No. MIR2250). Cell lysates from transfected HEK293T cells were analyzed by Western blot to confirm spike protein expression. Following confirmation, the mRNA was encapsulated in lipid nanoparticles as described below.

### mRNA-LNP formulation

All purified mRNAs generated above were encapsulated into lipid nanoparticles using the NanoAssemblr Benchtop system (Precision NanoSystems) and confirmed to have similar encapsulation efficiency (∼95%). In brief, mRNA was diluted in 50 mM sodium acetate buffer, pH 5.0 to achieve a working concentration of 0.096 mg/mL (Cayman Chemical, Cat. No. 35425). An ethanolic lipid mixture was prepared using four lipids—SM-102, 1,2-distearoyl-sn-glycero-3-PC, cholesterol, and DMG-PEG (Cayman, Cat. No. 35425) in a molar ratio of 50:10:38.5:0.38. Subsequently, diluted mRNA in an aqueous phase and lipid mixture was run through a microfluidic laminar flow cartridge (NanoAssemblr Ignite NxGen, Cat. No. NIN0061). This was done by maintaining a nitrogen-to-phosphate (N/P) ratio of 4.0 (Lipid mix to mRNA ratio of 4), an RNA-to-lipid flow ratio of 3:1, and a total flow rate of 12 mL/min to generate mRNA–lipid nanoparticles (mRNA-LNPs). The resulting mRNA-LNPs were concentrated and purified using an Amicon Ultra-15 filtration unit and a 0.2-µm Acrodisc filter. Encapsulation efficiency and the concentration of encapsulated mRNA were determined using the Quant-iT RiboGreen RNA Assay Kit (Invitrogen, Cat. No. R11490).

### Reagents, flow cytometry, and equipment

To determine the T-cell responses in the blood and spleen, single-cell suspensions of PBMCs and spleen were prepared as described previously (25). Dead cells were gated out using Live/Dead fixable dead cell stain (Invitrogen). The CD8 and CD4 responses specific to the OC43 spike were measured by stimulating splenocytes with the OC43 spike peptide pools (NR-53728, BEI) in intracellular cytokine staining (ICS). Biotinylated MHC class I monomer (K^b^S598, sequence RCQIFANI) was used for detecting MHV spike-specific CD8 T cells and was obtained from the NIH tetramer facility at Emory University. Cells were stained with fluorescently labeled antibodies against anti-mouse CD8α (53-6.7 on PerCP-Cy5.5), anti-mouse CD4 (RM4-5 FITC), anti-mouse CD44 (IM7 on Pacific Blue), anti-mouse IFNγ (XMG1.2 on APC), anti-mouse IL-2 (JES6-5H4 on PE), and anti-mouse TNFα (MP6-XT22 on PE/Cyanine7). Fluorescently labeled antibodies were purchased from BD Pharmingen, except for anti-CD44 (which was from Biolegend). Dead cells were gated out using LIVE/ DEAD fixable dead cell stain (Invitrogen). Flow cytometry samples were acquired with a Becton Dickinson Canto II or an LSRII and analyzed using FlowJo v10 (Treestar). The following reagent was obtained through BEI Resources, NIAID, NIH: Peptide Array, Human Coronavirus OC43 Spike (S) Glycoprotein, NR-53728.

### OC43 spike, HKU1 spike, SARS-CoV-2 spike, and MHV spike-specific ELISA

Binding antibody titers were measured using ELISA as described previously (26, 27). In brief, 96-well flat bottom plates MaxiSorp (Thermo Scientific) were coated with 0.1 mg/well of the respective spike protein for 24 h at 4°C. For detection of MHV spike-specific antibody responses, we utilized a lysate of HEK293T cells transfected with a plasmid encoding MHV spike, as coating antigen (incubated for 48 h at room temperature). Plates were washed with PBS + 0.05% Tween-20. Blocking was performed for 4 h at room temperature with 200 μL of PBS + 0.05% Tween-20 + bovine serum albumin. Then, 6 µL of sera was added to 144 μL of blocking solution in the first column of the plate, 1:3 serial dilutions were performed until row 12 for each sample, and plates were incubated for 60 min at room temperature. Plates were washed three times followed by the addition of goat anti-mouse IgG horseradish peroxidase-conjugated (Southern Biotech) diluted in blocking solution (1:5,000) at 100 µL/well and incubated for 60 min at room temperature. Plates were washed three times, and 100 µL/well of Sure Blue substrate (Sera Care) was added for approximately 8 min. The reaction was stopped using 100 µL/well of KPL TMB stop solution (Sera Care). Absorbance was measured at 450 nm using Spectramax Plus 384 (Molecular Devices). OC43 spike protein was produced in-house using a mammalian expression vector obtained from Addgene (Cat. No. 166015).

### Propagation and determination of OC43-NV titers

OC43-NV stocks were propagated in 1-2 days old suckling neonates from C57BL/6 mice using a protocol from a prior paper (28). In brief, ten 1-2-day-old mice were inoculated intracerebrally with 10 µL of brain homogenates infected with OC43-NV (kind gift from Dr. Stanley Perlman’s laboratory). After 2 days, whole brains were collected from the neonates and homogenized in 2 mL of sterile PBS. The lysates were clarified by centrifugation at 2,000 rpm for 10 min, as previously described (29), and small aliquots of the supernatant were stored in a −80°C. For adult mouse challenges, the above viral stock was diluted 10-fold, and 50 µL was administered intranasally. Viral load in brain lysates was quantified by quantitative real-time RT-PCR targeting the OC43 nucleocapsid gene, using TaqMan chemistry as previously described (20).

### OC43-NV challenge and disease severity score

On day 15 post-vaccination, mice were challenged intranasally with 50 µL of OC43-NV stock (1 × 10^10^ genome copies), administered as 25 µL per nostril. All mice were monitored for weight loss and clinical severity over the course of 3 weeks. Disease severity was measured in terms of clinical scores ranging from 0 to 3, defining the body posture, appearance of fur, eye secretions or closure, animal activity, lethargy, body temperature, and neurological symptoms (30, 31). The highest score was represented by severe disease status counting piloerection, puffy appearance, non-responsive and stationary even when provoked, severely hunched posture, completely closed eyes, stopped eating/drinking, rapid or labored breathing with gasps, cold body temperature, shivering, showing no response upon stimuli, and neurological symptoms. Score 2 was defined as moderate disease including moderately hunched posture, majority of fur on back is ruffled, active only when provoked, stationary, no response to auditory or slowed response to touch, eyes half-closed, potential eye secretions, lethargy, less active, and consistently labored breathing. The mild disease was scored as one showing mildly hunched, slightly ruffled fur, active, avoiding standing upright, and slowed response to auditory/touch stimuli. The normal active animal with a smooth coat was scored as zero.

### MHV propagation and quantification

Seed stock of MHV-A59 was obtained from ATCC. The virus was propagated in 17 CL-1 cells and tittered on L2 cells (kind gift from Dr. Susan R. Weiss). The 17 CL-1 and L2 cells were passaged in DMEM supplemented with 10% fetal bovine serum (FBS), 1% L-glutamine and 1% penicillin/streptomycin. For virus propagation, 17 CL-1 cells were inoculated at a low multiplicity of infection (MOI) of 0.1 in 1% DMEM. After 72 h of incubation, the supernatant was collected and clarified by centrifugation at 2,000 rpm for 10 min. The titer of the viral stock was determined by plaque assay using L2 cell monolayers. To determine the viral titer in infected tissues, the lung, liver, and brain were collected on day 3 after the challenge and stored immediately in a −80°C until processing. For plaque assay, 1 × 10^6^ L2 cells per well were seeded in 6-well plates in 10% DMEM medium. After 24 h, when the monolayer was 90%–00% confluent, tissue samples were thawed in a water bath at 37°C and processed to assess viral titer. The tissues were homogenized using a standard TissueRuptor homogenizer, and 10-fold serial dilutions of the homogenized samples were prepared in 1% DMEM and applied dropwise onto the cell monolayer. The six-well plates were placed in a 37°C, 5% CO_2_ incubator for 1 h and manually rocked every 10 min. After 1 h of incubation, a 1:1 agarose and 2 × 199 overlay was added to the monolayer, and plates were incubated at 37°C, 5% CO for 48 h. After 48 h, the overlay was removed, the cells were stained with 0.1% crystal violet, and plaques were counted.

### Adoptive plasma transfers

C57BL/6 donor mice were immunized with two doses of the mRNA-OC43 vaccine at a 3-week interval. Seven days after the final dose, OC43 spike-specific antibody responses were confirmed by ELISA, and plasma from the immunized mice was pooled. These pooled plasmas were adoptively transferred into C57BL/6 recipient mice via the i.p. route. Control mice received plasma from naïve donors. The following day, all mice were infected i.p. with 2 × 10⁶ PFU of MHV-A59. On day 3 post-infection, tissues were harvested and homogenized using a TissueRuptor Homogenizer (QIAGEN). Viral loads were quantified by plaque assay, as described above.

### Antibody treatments for CD4 and CD8 depletion

All antibodies used for *in vivo* treatments were purchased from BioXCell or Leinco, diluted in sterile PBS, and administered via intraperitoneal (i.p.) injection. CD4^+^ T cell-depleting antibody (GK1.5) and CD8^+^ T cell-depleting antibody (2.43) were given at a dose of 200 µg daily, starting 1 day before infection and continuing through day three post-infection. IgG isotype controls were included in all experiments as controls.

### Detection of IFN-γ-producing T cell responses via ELISpot

To detect IFN-γ-producing antigen-specific T cells, 96-well ELISpot plates (Millipore, Burlington, MA) were coated with anti-mouse IFN-γ monoclonal antibody (clone AN-18, BioLegend 517902) at 5  µg/mL and incubated overnight at 4°C. The following day, plates were washed twice with 200 µL/well of sterile 1× PBS and blocked with 200 µL/well of RPMI medium supplemented with 10% FBS, 1% L-glutamine, and 1% Pen/Strep for 2 h at 37°C in a CO_₂_ incubator. Single-cell suspensions from spleen or lymph nodes were prepared at 2.5 ×  10⁶ cells/mL in supplemented RPMI. After blocking, media were discarded, and plates were seeded with 2.5 ×  10⁵ cells/well and stimulated using 4 µg/mL of an OC43-spike peptide pools. Cells were incubated for 18–20 h at 37°C in a CO_₂_ incubator. Cells from naïve mice served as negative controls. For positive controls, cells were treated with either 10 ng/mL phorbol 12-myristate 13-acetate (PMA, Sigma) plus 500 ng/mL ionomycin (Sigma), or with anti-mouse CD3 and CD28 antibodies (1 µg/well). An equimolar concentration of DMSO was included as a negative control. Unstimulated controls included cells incubated with media alone. After 18–20 h of incubation, cells were discarded, and plates were washed five times with wash buffer (1× PBS + 0.05% Tween-20). Plates were then incubated for 90 min with biotinylated anti-IFN-γ antibody (clone R4-6A2, BioLegend 505704) at 0.5  µg/mL diluted in PBS with 10% FBS. After washing, streptavidin–alkaline phosphatase (Bio-Rad 170-6432), diluted 1:1000 in 10% PBS, was added and incubated for 45 min. Plates were washed with wash buffer and developed using substrate (Bio-Rad 170-6432) for 8 min. The reaction was stopped by rinsing plates with running water. Spot-forming cells (SFCs) were analyzed using an ImmunoSpot Image Analyzer (Cleveland, USA). The number of spot-forming units (SFU) per million cells was calculated as the mean of duplicate wells after subtraction of negative control wells (no antigen).

### Statistical analysis

Statistical tests used are indicated on each figure legend. Dashed lines in data figures represent the limit of detection. Statistical significance was established at a *P* value of 0.05 or less and was generally assessed by two-tailed unpaired Student’s t tests (Mann-Whitney *U* test), unless indicated otherwise in figure legends. Data were analyzed using Prism version 10 (GraphPad Software).

## Data Availability

All data supporting the findings of this study are included within the article. Requests for resources should be directed to the lead contact, Pablo Penaloza-MacMaster (ppenaloz@bidmc.harvard.edu).
